# Transcriptome profiling provides insights into the molecular mechanisms of maize kernel and silk development

**DOI:** 10.1186/s12863-021-00981-4

**Published:** 2021-08-21

**Authors:** Ting Li, Yapeng Wang, Yaqin Shi, Xiaonan Gou, Bingpeng Yang, Jianzhou Qu, Xinghua Zhang, Jiquan Xue, Shutu Xu

**Affiliations:** Key Laboratory of Biology and Genetic Improvement of Maize in the Arid Area of Northwest Region, College of Agronomy, Northwest A&F University, Yangling, 712100 Shaanxi Province China

**Keywords:** Transcriptome, Kernels, Silks, Pollination, Maize

## Abstract

**Background:**

Maize kernel filling, which is closely related to the process of double fertilization and is sensitive to a variety of environmental conditions, is an important component of maize yield determination. Silk is an important tissue of maize ears that can discriminate pollen and conduct pollination. Therefore, investigating the molecular mechanisms of kernel development and silk senescence will provide important information for improving the pollination rate to obtain high maize yields.

**Results:**

In this study, transcript profiles were determined in an elite maize inbred line (KA105) to investigate the molecular mechanisms functioning in self-pollinated and unpollinated maize kernels and silks. A total of 5285 and 3225 differentially expressed transcripts (DETs) were identified between self-pollinated and unpollinated maize in a kernel group and a silk group, respectively. We found that a large number of genes involved in key steps in the biosynthesis of endosperm storage compounds were upregulated after pollination in kernels, and that abnormal development and senescence appeared in unpollinated kernels (KUP). We also identified several genes with functions in the maintenance of silk structure that were highly expressed in silk. Further investigation suggested that the expression of autophagy-related genes and senescence-related genes is prevalent in maize kernels and silks. In addition, pollination significantly altered the expression levels of senescence-related and autophagy-related genes in maize kernels and silks. Notably, we identified some specific genes and transcription factors (TFs) that are highly expressed in single tissues.

**Conclusions:**

Our results provide novel insights into the potential regulatory mechanisms of self-pollinated and unpollinated maize kernels and silks.

**Supplementary Information:**

The online version contains supplementary material available at 10.1186/s12863-021-00981-4.

## Background

Maize (*Z. mays* L.), which has been domesticated over the past ~ 10,000 years, is one of the most important cereal crops worldwide and is grown for use as food, feed and raw material for industry [1, 2]. As a result of breeding progress and artificial selection, the yield of maize has increased extensively over the past hundred years, but further increases are needed to meet the demands imposed by the rapid development of industrialized economies [3]. Pollination, an important and complex process with significant effects on in vivo kernel setting and yield stability, is sensitive to the impacts of floral morphology [4] and water [5, 6], nitrogen [7] and other environmental conditions [8]. Nevertheless, the poor understanding of the regulatory mechanisms underlying pollination is an impediment to improving the pollination rate and thereby increasing yield in maize.

Maize is a monoecious plant with unisexual male and female flowers, and floral development in maize is illustrated using the ABCDE model [9, 10]. The male inflorescence (named tassel) is located in the apex of the plant and generates abundant pollen. The female inflorescence (named ear) is located within the axial vegetative leaves and produces silks [10–12]. In the maize pollination period, mature pollen grains are released from anthers in the tassel and drop onto the surface of silks. Then, compatible pollen grains hydrate, germinate, and produce pollen tubes that grow down to the ovules, with complete fertilization following [13]. During the pollination process, maize silks play a vital role in accepting pollen grains for the completion of fertilization, but there are few studies on this topic. Maize kernel filling plays an important role in maize yield determination and mainly involves the conversion of imported sucrose and amino acids into starch and storage proteins in the endosperm [14–16]. The developmental pattern of double fertilization has been extensively studied [4, 17]. After double fertilization, the zygote begins to undergo asymmetric cell division to form progenitors of the embryo and endosperm. Then, after further cell division, cell expansion and endoreduplication, the embryo and endosperm enlarge significantly. Genetic studies have identified a large number of genes involved in key steps of the regulation of embryogenesis and the biosynthesis of endosperm storage compounds, such as *opaque2* (*o2*) [18], *Shrunken2* [19], and *knotted1* (*kn1*) [20]. However, little is known about the developmental pattern of uncompleted double-fertilized maize ovules, which are known as unpollinated kernels (KUP).

In high-throughput RNA sequencing (RNA-seq) experiments, the total mRNA of collected samples can be extracted and sequenced to determine expression levels of genes. Recently, high-throughput transcriptomic approaches have proven powerful for studying the regulatory networks of cereal kernel development [16, 17, 21]. With the development of RNA-seq technology, the detection of transcript expression levels and particular structures has become more precise, and several pipelines for RNA-seq analysis have been developed [22–24]. Meanwhile, the reference maize B73 genome has been improved to version 5 (https://www.maizegdb.org/, Zm-B73-reference-NAM-5.0), which is more complete and accurate than previous versions. The improved version has accelerated the application of genomics and transcriptomics in the genetics and molecular biology fields. In this study, we selected HISAT-StringTie-DESeq2 as an RNA-seq analysis pipeline and conducted RNA-seq to analyse transcript changes in self-pollinated and unpollinated maize ear tissues (kernels and silks) using the maize B73 version 5.0 genome as a reference. Our objective was to preliminarily explore the molecular mechanisms of maize kernel and silk development through the identification, exploration and annotation of differentially expressed transcripts (DETs) between self-pollinated and unpollinated maize kernels and silks, and the analysis of transcription factors (TFs). Ultimately, this work will provide new insights into the molecular mechanisms of maize kernel and silk development.

## Results

### Overview of RNA-seq data

**Fig. 1:**
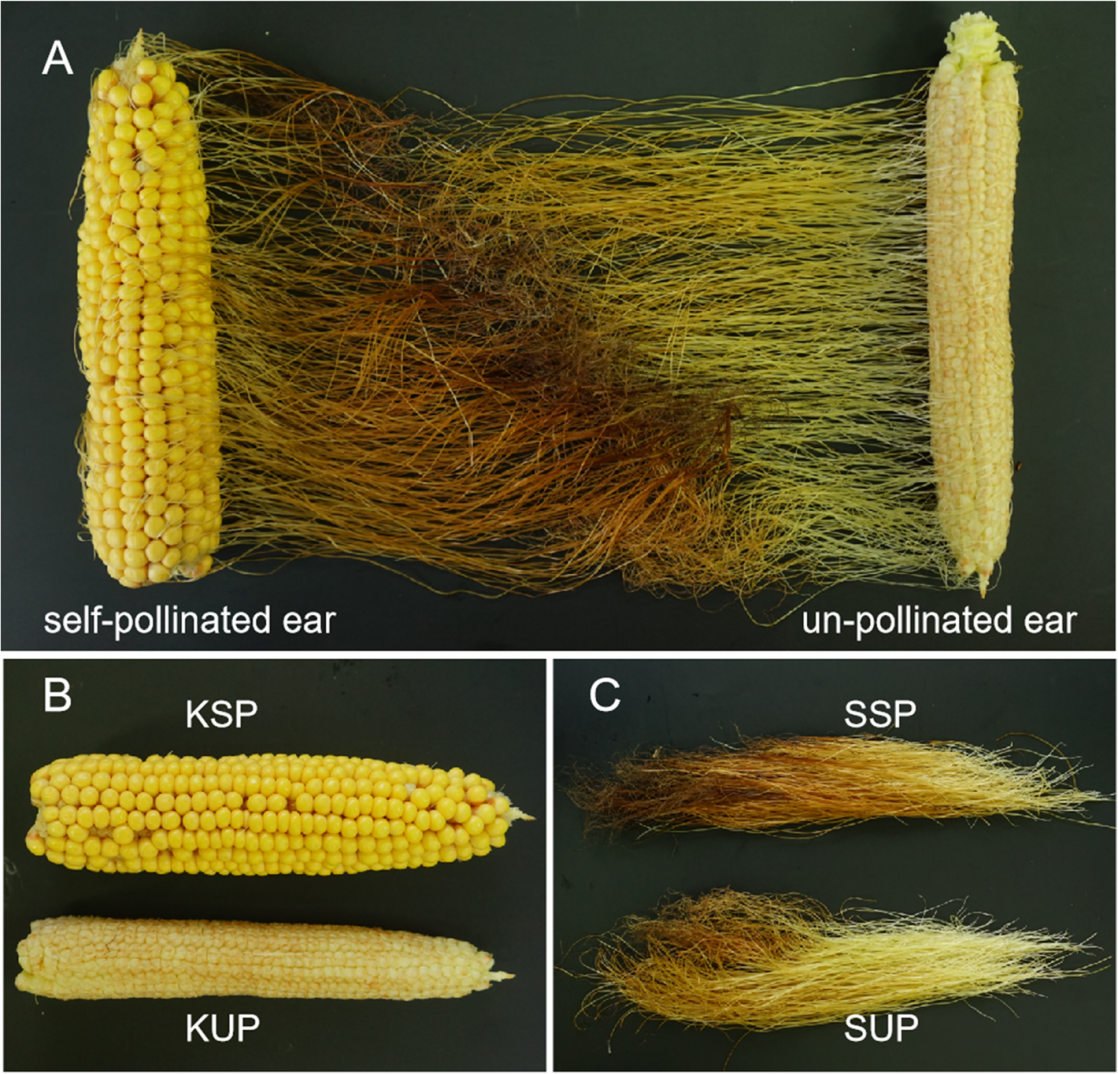
Phenotypes of self-pollinated and unpollinated ears. **A** Comparisons of self-pollinated and unpollinated ears, **B** self-pollinated and unpollinated kernels, and **C** self-pollinated and unpollinated silks

**Fig. 2:**
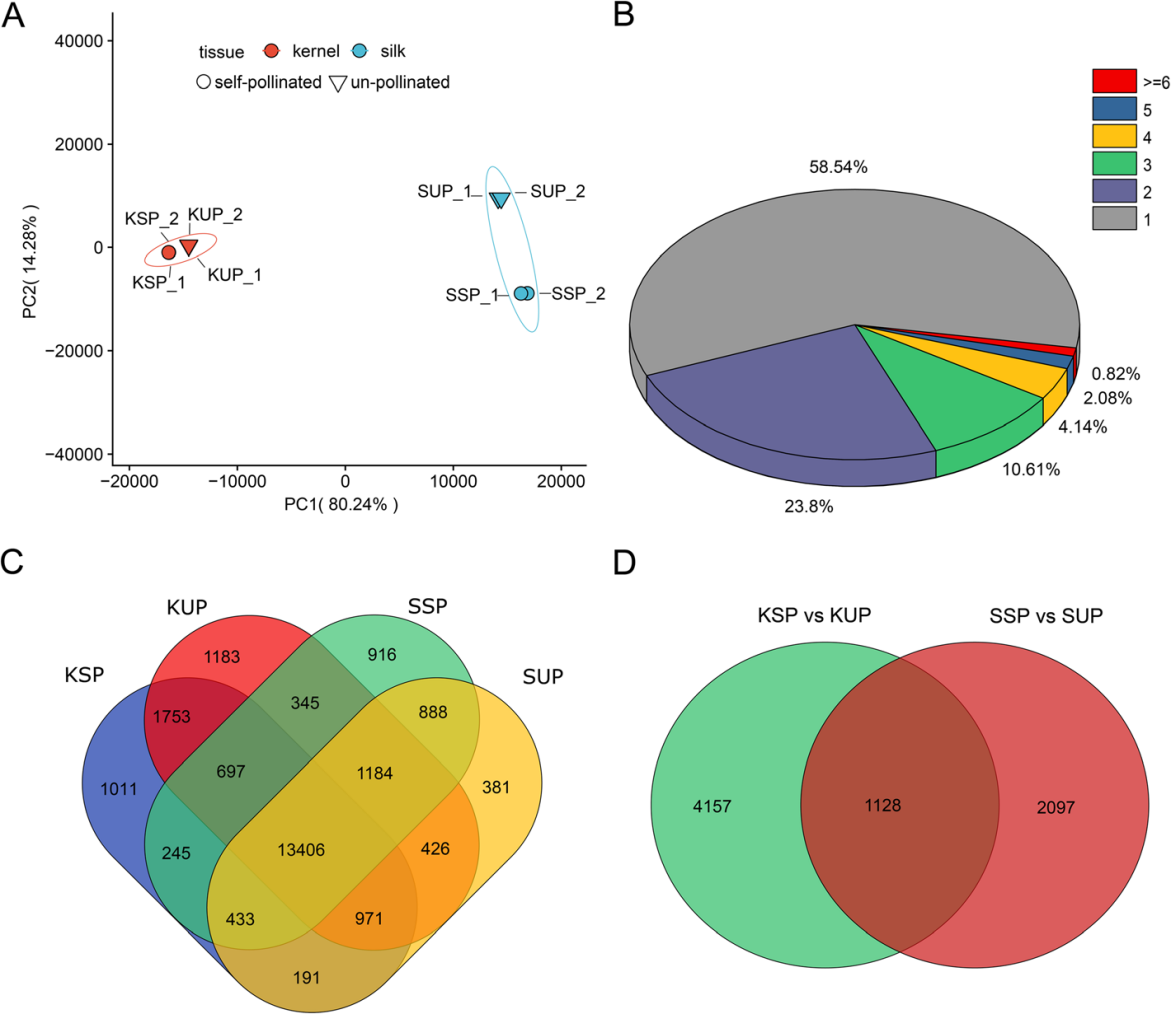
Summary of the RNA-seq data from this study. **A** The PCA plot for all samples. **B** Proportions of transcripts detected from different genes in all samples. **C** Venn diagram of exclusively detected transcripts. **D** Venn diagram of differentially expressed transcripts in kernels and silks

**Fig. 3:**
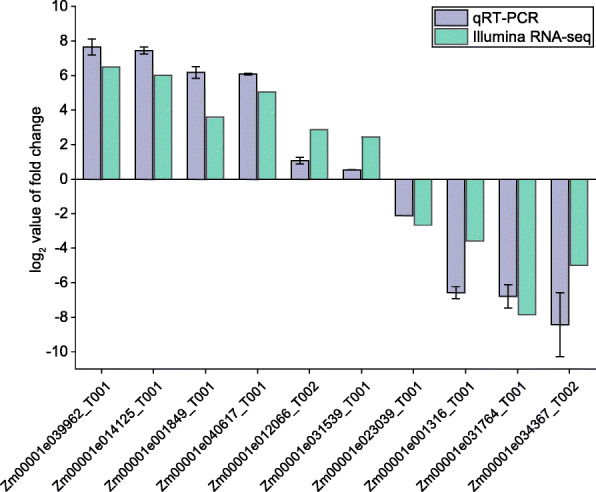
Quantitative RT-PCR (qRT-PCR) validation of differentially expressed genes (DETs) between self-pollinated and unpollinated maize ears. Error bars indicate standard error (SE) of three biological replicates

In this study, self-pollinated and unpollinated maize ear tissues (kernel and silk; KSP, KUP, SSP and SUP) were selected for RNA sequencing with two biological replicates (Fig. 1). Initially, a total of 215.62 Gb of raw data were obtained after completing the paired-end sequencing protocol. We filtered the raw reads according to a quality score of less than 20 and removed adaptor sequences with fastp software. The Q-scores for more than 97.44% of the reads were Q20, and 36,731,405 ~ 43,427,959 paired reads were obtained for each group of samples (Table 1). We mapped these clean reads to maize reference genome sequences using the HISAT2 procedure. Among the mapped reads, divergent alignment rates were observed between the kernel and silk samples: the kernel samples showed rates of 78.48% ~ 81.79%, and the silk samples showed rates of 49.16% ~ 61.22%. In particular, the SSP alignment rate was 49.16% ~ 49.47%, which may have been the result of senescence in the silks; these results are consistent with previous reports [25–27]. After the completion of the HISAT-StringTie-Ballgown analysis pipeline, the FPKM value matrix was obtained with the default stringent criteria of Ballgown. As shown in Fig. 2A, the two biological replicates of each sample were strongly correlated, and only one transcript was detected for most genes (58.54%) (Fig. 2B). To validate the differential expression results from our transcriptome sequence data analysis, the expression of 10 randomly selected DETs with only one transcript was evaluated by qRT-PCR. The selected transcripts included WRKY transcription factor 74, a gibberellin receptor GID1L2 precursor, asparagine synthetase3, sweet4c and others (Fig. 3, Additional file 1). The expression levels determined by qRT-PCR were in agreement with the changes in transcript abundance determined by RNA-seq analysis, which suggested that our transcriptome profiling data were highly reliable.

**Table 1:** Summary of RNA-seq data alignments for KSP, KUP, SSP and SUP

Sample	Q20 percentage (%)	Paired reads	Alignment rate (%)	Aligned exactly 1 time (%)
KSP_1	97.76	42,799,164	81.79	78.37
KSP_2	97.44	43,427,959	81.49	78.08
KUP_1	97.65	38,2965,90	78.48	75.67
KUP_2	97.60	36,731,405	78.61	75.77
SSP_1	97.74	37,176,533	49.16	47.09
SSP_2	98.00	38,301,583	49.47	47.39
SUP_1	97.73	41,794,608	61.22	59.08
SUP_2	97.56	40,972,293	60.93	58.82

### Transcriptional changes in self-pollinated and unpollinated maize ear tissues

To perform a more detailed analysis of the transcriptional changes in self-pollinated and unpollinated maize ear tissues, genes with low expression (FPKM ≤1) were filtered. In total, 24,030 transcripts (20.36% of all maize transcripts) were detected in all samples (Additional file 2), 13,406 transcripts were common to more than one group, and 1011, 1183, 916 and 381 transcripts were exclusively detected in KSP, KUP, SSP and SUP, respectively (Fig. 2C). Subsequently, we annotated the transcripts exclusively detected in KSP, KUP, SSP or SUP. Notably, we found that a large number of transcripts associated with starch biosynthetic and metabolic processes and carbohydrate metabolism showed enrichment in KSP, whereas in KUP, most of the transcripts detected exclusively in these kernels were related to plant-type cell wall organization. In silks, transcripts associated with the regulation of unidimensional cell growth and pollen tube growth were detected in SSP, but only xyloglucosyl transferase activity, which is important in maintaining cell homeostasis, was obtained for SUP (Additional file 3).

### Functional characterization of DETs in self-pollinated and unpollinated maize ear tissues

**Fig. 4:**
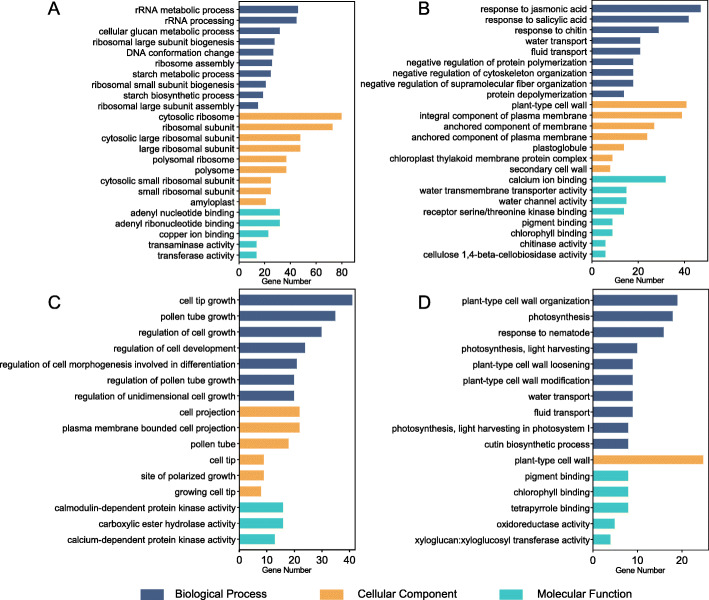
The most significantly enriched Gene Ontology (GO) annotations of DETs in self-pollinated and unpollinated maize ear tissues. **A** The most enriched GO terms of upregulated KSP DETs. **B** The most enriched GO terms of downregulated KSP DETs. **C** The most enriched GO terms of upregulated SSP DETs. **D** The most enriched GO terms of downregulated SSP DETs. The horizontal axis indicates the number of each GO term present in the DET dataset

**Fig. 5:**
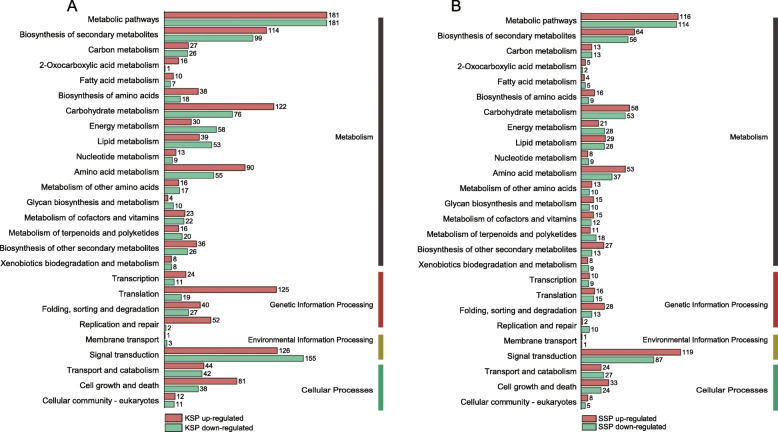
Kyoto Encyclopedia of Genes and Genomes (KEGG) functional classification of DETs between self-pollinated and unpollinated maize ears. **A** Self-pollinated and unpollinated kernels. **B** Self-pollinated and unpollinated silks. Values beside the bars represent the numbers of components in each pathway present in the DET dataset

To obtain a complete list of the DETs from self-pollinated and unpollinated maize ear tissues, we selected the prepDE Python script to obtain transcript count matrices. DESeq2 with stringent criteria (log_2_FC > 2 or log_2_FC < − 2 and p.adj < 0.05) was used to identify significant DETs. As a result, 5285 and 3225 DETs were determined to be significantly differentially expressed in the kernel (KSP vs KUP) and silk (SSP vs SUP) groups, respectively (Fig. 2D). Relative to KUP, there were 2331 upregulated DETs and 2954 downregulated DETs in KSP, and relative to SUP, there were 1782 upregulated DETs and 1443 downregulated DETs in SSP (Additional file 4). To explore which biological processes play key roles after pollination in kernels and silks, GO enrichment analysis of DETs was carried out. In the kernel groups (KSP vs KUP), a large number of upregulated KSP DETs were enriched in starch biosynthetic and metabolic processes, cellular glucan metabolic processes, and rRNA metabolic processes. More importantly, the most enriched cellular component categories of the upregulated KSP DETs were the cytosolic ribosome and amyloplast (Fig. 4A). However, a large number of downregulated DETs from KSP showed annotations related to the responses to jasmonic acid, salicylic acid and chitin, and the mainly enriched cellular component category of downregulated DETs from KSP was plant-type cell wall (Fig. 4B). Additionally, in the kernel group, we found a large number of DETs involved in metabolism, genetic information processing, environmental information processing and cellular processes, and more KEGG pathways related to metabolism were associated with the upregulated KSP DETs than with the downregulated KSP DETs; these pathways included the biosynthesis of amino acids, carbohydrate metabolism and fatty acid metabolism, among others (Fig. 5A). More strikingly, more KEGG pathways involved in transcription, translation, cell growth and death, DNA replication and repair were associated with the upregulated DETs in KSP. These results are consistent with the development of kernels.

We performed GO and KEGG pathway enrichment analyses of the silk groups to explore the biological processes that play key roles after pollination. According to the GO enrichment analysis results, the regulation of cell growth and cell tip growth, pollen tube growth and the regulation of unidimensional cells were annotated among the upregulated SSP DETs, and the most enriched cellular component categories among the upregulated SSP DETs were pollen tubes and cell projections (Fig. 4C). However, photosynthesis, cutin biosynthetic process and plant-type cell wall organization were annotated categories among the downregulated SSP DETs (Fig. 4D). In addition, we found a large number of metabolic, genetic information processing, environmental information processing and cellular process KEGG pathways among the silk DETs. Pathways involved in protein folding, sorting and degradation were more enriched among the upregulated SSP DETs, whereas pathways involved in DNA replication and repair were more enriched among the downregulated SSP DETs (Fig. 5B).

Notably, the results of the enrichment analysis of DETs were similar to those obtained for transcripts exclusively detected in kernels and silks, with enrichment detected for categories such as starch biosynthetic and metabolic processes, the regulation of unidimensional cell growth, and pollen tube growth (Fig. 4, Additional file 3). This similarity may be explained by the fact that most of the DETs were derived from genes expressed specifically in different stages of kernels and silks.

### Expression levels of senescence- and autophagy-related genes

Researchers have found that preventing pollination induces the initiation of senescence in maize leaves, which is always accompanied by cell death [28]. According to the enrichment analysis results, the DETs from kernels and silks were significantly enriched in cell growth and death pathways. Therefore, we focused on the expression of senescence-related genes in self-pollinated and unpollinated maize ear tissues. In the public leaf senescence database (https://bigd.big.ac.cn/lsd/), we observed 70 senescence genes with genomic sequences corresponding to 134 transcripts in maize. Among these genes, 60 genes (71 transcripts) were expressed in maize kernels and silks (Additional file 5). Moreover, the expression levels of senescence genes were clearly different between maize kernel and silk tissues, and more senescence genes were highly expressed in maize silks than in maize kernels.

Autophagy is a primary intracellular degradation pathway that contributes to nutrient recycling and is related to senescence [29]. To observe the expression levels of autophagy-related genes, we obtained 40 autophagy-related genes corresponding to 109 transcripts from the public database and determined the expression of these genes in maize ear tissues. As shown in Additional files 6, 35 autophagy-related genes (57 transcripts) were expressed in maize kernels and silks. Similar to the senescence-related genes, the majority of autophagy-related genes exhibited higher expression in maize silks than in kernels. Overall, these results indicate that the expression of autophagy-related genes and senescence-related genes is prevalent in maize kernels and silks and that pollination changes the expression levels of several senescence-related and autophagy-related genes in maize kernels and silks.

### Identification of TFs associated with maize ear tissue development

**Fig. 6:**
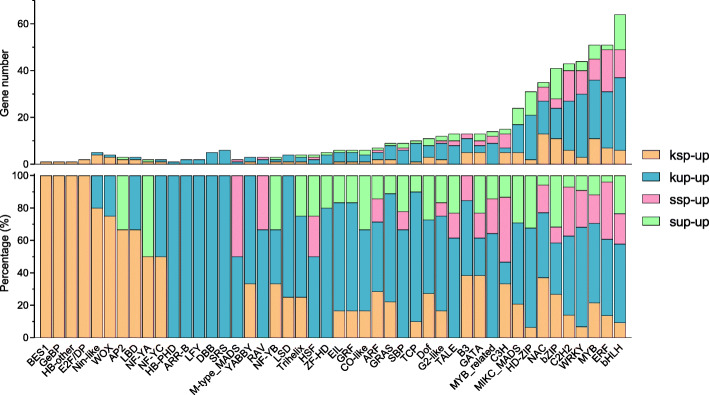
Numbers and percentages of upregulated TFs in different tissues: we calculated the percentage of upregulated TFs in each tissue/all upregulated TFs

The regulation of gene expression is essential for plant growth and development, governing the perception and responses of plant cells and tissues to different stimuli. TFs are key genomic regulatory elements that play crucial roles in regulating the expression of related genes. Here, we extracted TFs from the DETs to explore the expression of a common set of TF families in self-pollinated and unpollinated maize ear tissues. Strikingly, a total of 518 differentially expressed TFs from 46 families were identified in the four maize ear tissues (Additional file 7), and several small TF family genes were found to be upregulated in one of the tissues (Fig. 6), which implied that they may play a role in the development of only one of the four tissues examined in our study. Notably, the TF family members with high relative expression differed among different tissues. As shown in Fig. 6, most TF family members, such as bHLH, C2H2, ERF, GRAS, GRF, TCP, and WRKY TFs, were highly expressed in KUP tissues relative to the other studied tissues, but some TF family members, such as C3H, and GATA TFs, were highly expressed in KSP, SSP and SUP tissues.

## Discussion

In the present study, we performed RNA-seq to explore the molecular mechanisms underlying development in self-pollinated and unpollinated maize kernels and silks, and the most recent and accurate reference genome of maize B73 was used to map clean paired-end reads. Our results showed that at least 18,158 genes with 24,030 transcripts were required to program maize kernel and silk development. Over 40% of the genes produced more than two transcripts during this process. Global comparisons of gene expression highlighted significant changes in the gene expression patterns of kernels and silks after pollination. We also found some specific genes and TFs that were highly expressed in only one of the studied tissues.

### Genes involved in the development of KSP and KUP

Large-scale gene expression analyses can be implemented through GO and KEGG pathway enrichment analyses, and the integration of these analyses can provide abundant information about the regulation of gene networks at the cellular level. In maize, upon the completion of pollination, the embryo and endosperm begin to form gradually in normal kernels, and sucrose and amino acids are quickly converted into starch and storage proteins in the endosperm, with the starch accumulation rate reaching a maximum at 20 DAP [16]. According to the results of GO and KEGG enrichment analyses, a large number of DETs involved in starch biosynthetic and metabolic processes and carbohydrate metabolism were highly expressed in KSP. Starch is the main component of mature maize kernels, and starch content is one of the main targets of maize breeding. Starch biosynthesis requires the proper execution of a series of coordinated enzymes [30], and the enzymes ADP-glucose pyrophosphorylase (AGPase), soluble starch synthase (SSS) and starch branching enzyme (SBE) are the major enzymes that catalyse starch biosynthetic substrate production and starch chain elongation and branching [30]. The starch synthase gene *zSSIIa* has been shown to encode granule-bound starch synthase I (also called Waxy protein) in the maize endosperm. Our results showed that *zSSIIa* was highly expressed in KSP, whereas it showed almost no expression in KUP, SSP and SUP (Additional file 8). *Zmdull1* encodes a starch synthase, most likely starch synthase II, which plays a determinant role in the biosynthesis of endosperm starch; it was highly expressed in KSP, similar to *zSSIIa*. AGPase provides ADP-glucose as the glucosyl donor for starch synthesis, and *Brittle2* (*Bt2*) encodes a characteristic AGPase in maize endosperm starch biosynthesis [7]. In this study, the expression of this gene was not detected in any sample, but another AGPase gene, *Shrunken2,* was highly expressed in KSP [31], whereas it showed almost no expression in KUP, SSP or SUP. In addition, there were other genes involved in the starch biosynthesis pathway that were highly expressed in KSP, such as *Shrunken1*, *sucrose synthase-1* (S*us1*), and *starch branching enzyme1* (*Sbe1*) [17].

In maize, the seeds store most amino acids as proteins rather than as free amino acids in the endosperm. Zeins are the most important seed storage proteins in maize endosperm and can be divided into α, β, γ, and δ zeins [32]. Previous research has confirmed that there are 30 α, 1 β, 3 γ, and 1 δ zein genes in B73 bacterial artificial chromosomes (RefGen_v2) and that three-quarters of zein genes are highly expressed in endosperm [17]. Maize o2 makes a large contribution to the synthesis of a 22-kD α-zein protein and several other zein proteins in maize endosperm, and mutations of the *o2* gene result in small, unexpanded protein bodies in maize endosperm [33]. In this study, we found that the maize *o2* gene was highly expressed only in KSP and that zein genes were significantly upregulated in KSP. These results provide preliminary evidence that the expression pattern of zein genes might be regulated by *o2*. In KUP, genes involved in starch and zein biosynthesis showed low or no expression, whereas genes involved in senescence and autophagy showed relatively high expression. In maize B73 leaves, free glucose and starch accumulation, chlorophyll loss and senescence have been shown to be significantly triggered by the prevention of pollination [34]. In this study, the highly expressed DETs in KUP were enriched in GO categories related to the responses to jasmonic acid, salicylic acid and chitin, and a large number of TFs were highly expressed. Likewise, we found plant-specific TCP TF family members containing a basic helix-loop-helix (bHLH) TCP domain that were highly expressed in the KUP samples [35]. In plants, jasmonic acid, salicylic acid and chitin are related mainly to plant disease and insect resistance [36], and in maize leaves, jasmonic acid is known to promote senescence mainly through the degradation of chlorophyll [37]. TCP proteins mainly regulate plant proliferation and cell division, and the overexpression of several *TCP* genes can disturb plant tissue development [38, 39]. Considering these findings together, we speculate that abnormal development and senescence appeared in KUP and that a larger number of TFs were specifically expressed to regulate kernel development.

### Genes involved in the regulation of SSP and SUP

Silks anchored to each ovule are a necessary component of maize ears, and the function of silks is equivalent to that of the stigma in typical flowering plants in supporting pollen hydration and germination. After pollen grain hydration, the silks form a pollen tube that penetrates the cortical parenchyma and reaches the ovule [40]. In this study, a large number of DETs involved in the regulation of cell growth and cell tip growth, pollen tube growth and the regulation of unidimensional cells were annotated in the group of upregulated SSP DETs. This implies that maize pollination is complete but that pollen tube growth is ongoing. The nutrient-rich, fluid-filled properties of maize silks facilitate their fertility-related functions but simultaneously make the tissue vulnerable to most maize fungal pathogens and insect pests [40]. A maize glycine-rich protein (*ZmGRP5*) has been identified to function in maintaining the structure of silks [40]. We found that this gene appeared to be relatively highly expressed in SSP and SUP, especially in SSP. Previous studies showed that two types of TFs function in maintaining the development of maize silks (*ZmP1*/*ZmP2* and *ZmbZIP25*) [41, 42]. Our results indicated that these two types of TFs were highly expressed in SSP and SUP. Moreover, we found additional TFs that were highly expressed in SSP and SUP, such as MYBs and WRKYs; the functions of these TFs in maize silk development are unclear and require further study. Additionally, we found different genes related to senescence and autophagy that were highly expressed in SSP and SUP and even in kernels. The detailed mechanisms of senescence and autophagy during pollination need to be further investigated in the future.

## Conclusions

In this study, RNA-seq analysis was conducted to investigate the development of self-pollinated and unpollinated maize kernels and silks. We found that a large number of genes involved in key steps of the biosynthesis of endosperm storage compounds were upregulated after pollination. Furthermore, abnormal development and senescence appeared in KUP. We also identified several genes with functions in maintaining the structure of silks that were highly expressed in silks. In addition, we identified various TFs expressed in self-pollinated and unpollinated maize kernels and silks, especially in KUP. This large collection of genes provides a rich resource for future maize kernel and silk development studies, which will greatly enhance our understanding of the genetic control of early seed development in maize.

## Methods

### Plant materials and growth conditions for field experiments

KA105, a superior female inbred line of two nationally approved commercial hybrids (SD650: No.20200261; SD620: No.20200264), was selected from the Shaan A group cultivated by Northwest A&F University [43]. The plants were cultivated at approximately 67,500 plants/ha in summer in Yangling, Shaanxi Province, China. Field experiments were carried out under normal field management without water or nutrient stress. The ears were bagged with Kraft paper bags before the silking stage. After silking, several plants were manually self-pollinated, whereas others were left bagged. Two biological replicates of self-pollinated and unpollinated ears were harvested 20 days after pollination (DAP). Then, kernels and silks were separated from maize ears, frozen in liquid nitrogen and stored at − 80 °C until RNA extraction. The examined samples were referred to as self-pollinated kernels (KSP), unpollinated kernels (KUP), self-pollinated silks (SSP) and unpollinated silks (SUP).

### RNA isolation, cDNA library construction and RNA-seq

Total RNA from all samples was extracted with an RNA Sample Total RNA Kit (Tiangen, China), and a 2100 Bioanalyzer was then used to evaluate RNA quality. Qualified RNA samples were digested with DNase I (Takara, Japan) at 37 °C for 30 min. Dynabeads Oligo (dT) 25 (Life, USA) was used for mRNA purification. Sequencing libraries were constructed according to the manufacturer’s instructions of the employed NEBNext Ultra™ RNA Library Prep Kit for Illumina (NEB, USA). Then, the generated libraries were sequenced on the Illumina HiSeq 2500 platform to generate 125 bp short paired-end reads.

### RNA-seq data analysis

Fastp software was used to obtain clean reads by trimming adaptor sequences and removing low-quality reads (quality score < 20) from the raw reads [44]. Then, clean reads were mapped to the maize genome sequence (B73_RefGen_v5, https://www.maizegdb.org/) with HISAT2, and SAM files were sorted and converted to BAM files using SAMtools. StringTie was utilized to assemble transcripts and to estimate the expression of the transcripts based on the BAM files. The fragments per kilobase of transcript per million fragments mapped reads (FPKM) value matrix of all samples was extracted with the R package Ballgown. For differential expression analysis, we used the prepDE Python script to obtain transcript count matrices, and DESeq2 with stringent criteria (log_2_FC > 2 or log_2_FC < − 2 and p.adj < 0.05) was used to confirm significant differences in transcript expression (DETs). The sequence data from this study can be found in the NCBI Sequence Read Archive under BioProject ID PRJNA745969.

### TF and transcript function analysis

The maize TF list was retrieved from PlantTFDB (http://planttfdb.cbi.pku.edu.cn). To analyse the potential functions of the proteins corresponding to the obtained transcripts, we first reannotated all maize proteins. Briefly, all maize (B73_RefGen_v5) proteins were functionally annotated according to an updated list of Gene Ontology (GO) terms, Kyoto Encyclopedia of Genes and Genomes (KEGG) pathways, Pfam/SMART domains and Clusters of Orthologous Groups (COG) functional categories by using eggnog-mapper to build org.Zmays.eg.db [45]. The R package clusterProfiler was used to identify enriched GO terms with a cut-off of *P*-value < 0.05 [46]. For KEGG pathway analysis, the amino acid sequences of downregulated and upregulated transcripts were uploaded to BlastKOALA (https://www.kegg.jp/blastkoala/), and BLASTP analysis was then performed to obtain potential KEGG pathways.

### Quantitative reverse transcription RT-PCR analysis (qRT-PCR)

Approximately 10 DETs identified among the two groups of samples were verified by qRT-PCR performed on a QuantStudio 7 Flex Real-Time PCR System (Thermo Fisher, America) with a SuperReal PreMix Plus kit (SYBR Green) (Tiangen, China). Maize *tubulin* was used as an internal reference gene to normalize the relative expression of randomly selected DETs. Gene-specific primers were designed based on maize gene nucleotide sequences using Primer 5.0 (Additional file 1). All qRT-PCR experiments were performed using three biological replicates. The relative expression levels of each gene were calculated using the 2^-∆∆CT^ method in comparison with the control.

## Supplementary Information

**Additional file 1: Table S1.** Primers used for qRT-PCR in this study.

**Additional file 2: Table S2.** Matrix of filtered transcript expression.

**Additional file 3: Table S3.** GO annotations of transcripts exclusively detected in KSP, KUP, SSP and SUP.

**Additional file 4: Table S4.** Analysis of differentially expressed transcripts between kernels and silks.

**Additional file 5: Figure S1.** Expression levels of senescence-related genes in KSP, KUP, SSP and SUP. The color scale represents the normalized FPKM values (blue indicates lower expression, red indicates higher expression).

**Additional file 6: Figure S2.** Expression levels of autophagy-related genes in KSP, KUP, SSP and SUP. The color scale represents the normalized FPKM values (blue indicates lower expression, red indicates higher expression).

**Additional file 7: Table S5.** Average expression of differentially expressed TFs.

**Additional file 8: Table S6.** Genes involved in the development of self-pollinated and unpollinated kernels and silks.

## Data Availability

The datasets generated and/or analysed during the current study are available in the NCBI Sequence Read Archive (RNA sequencing data: BioProject PRJNA745969, https://dataview.ncbi.nlm.nih.gov/object/PRJNA745969).

## Abbreviations

DETs: Differentially expressed transcripts
TFs: Transcription factors
RNA-seq: RNA sequencing
KUP: Unpollinated kernels
KSP: Self-pollinated kernels
SUP: Unpollinated silks
SSP: Self-pollinated silks
20 DAP: 20 days after pollination
FPKM: Fragments per kilobase of transcript per million fragments mapped reads
GO: Gene Ontology
KEGG: Kyoto Encyclopedia of Genes and Genomes
COG: Clusters of Orthologous Groups
qRT-PCR: quantitative reverse transcription PCR
