# Supplementary figures and images for: Transcriptome profiling provides insights into the molecular mechanisms of maize kernel and silk development
Source: BMC Genom Data. 2021 Aug 21;22:28. doi: 10.1186/s12863-021-00981-4 (PMC8379809; doi:10.1186/s12863-021-00981-4)

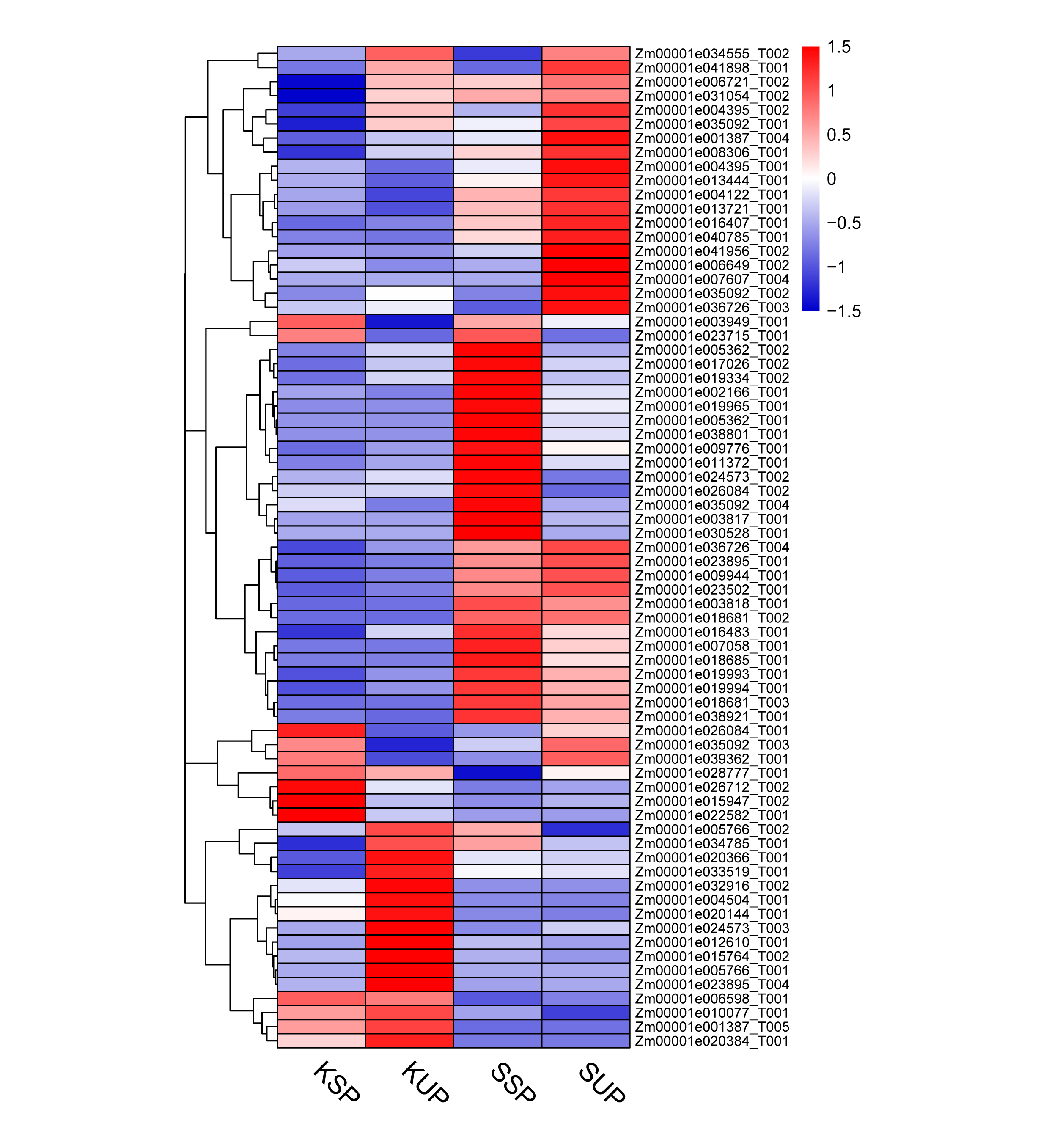

Supplement: Supplementary file 5 — Additional file 5: Figure S1. Expression levels of senescence-related genes in KSP, KUP, SSP and SUP. The color scale represents the normalized FPKM values (blue indicates lower expression, red indicates higher expression). [file 12863_2021_981_MOESM5_ESM.tif]

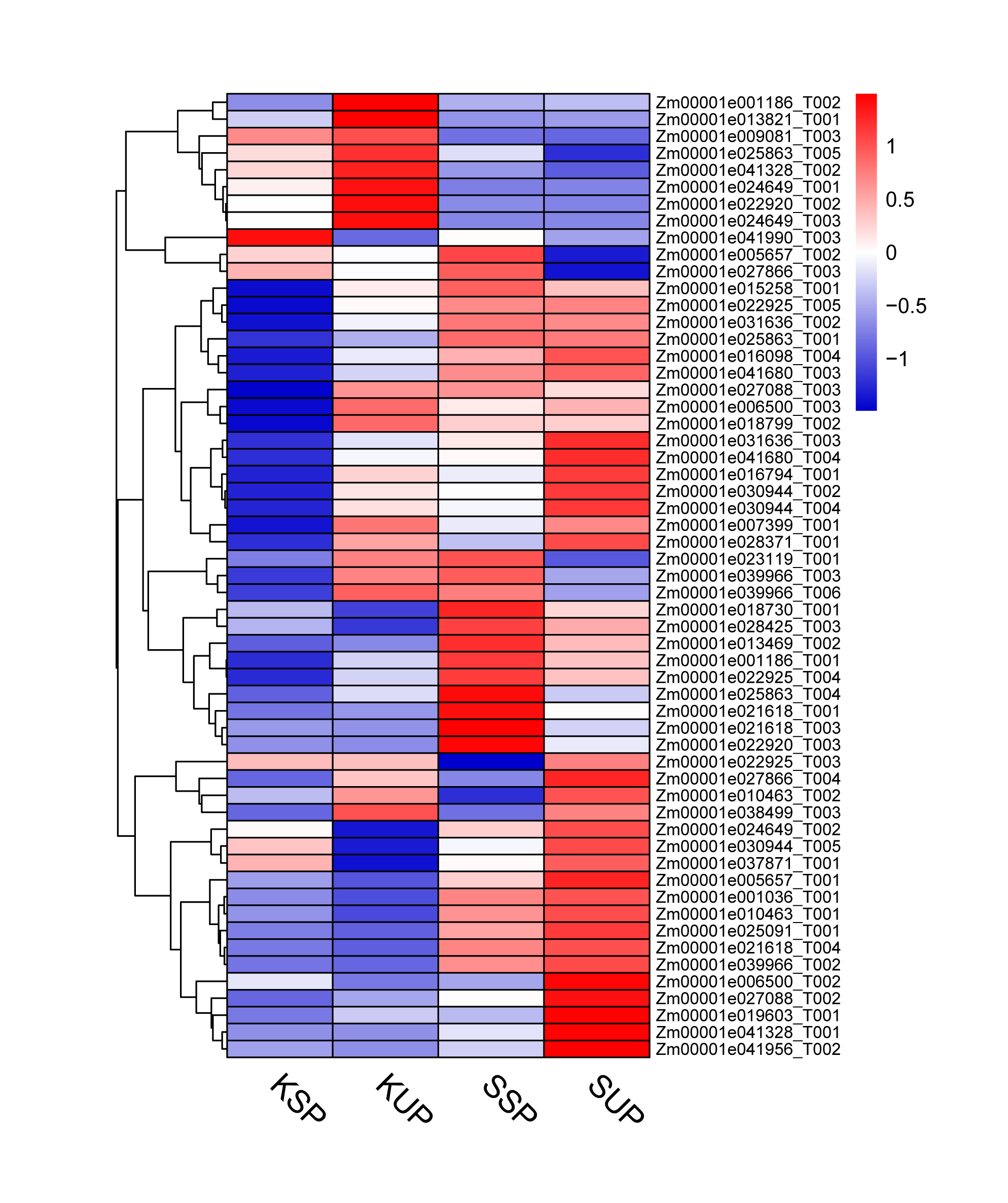

Supplement: Supplementary file 6 — Additional file 6: Figure S2. Expression levels of autophagy-related genes in KSP, KUP, SSP and SUP. The color scale represents the normalized FPKM values (blue indicates lower expression, red indicates higher expression). [file 12863_2021_981_MOESM6_ESM.tif]
